# Mcl-1 dynamics influence mitotic slippage and death in mitosis

**DOI:** 10.18632/oncotarget.6894

**Published:** 2016-01-12

**Authors:** Olivia Sloss, Caroline Topham, Maria Diez, Stephen Taylor

**Affiliations:** ^1^ Faculty of Life Sciences, University of Manchester, Manchester M13 9PT, United Kingdom; ^2^ Present Address: School of Environment & Life Sciences, Cockcroft Building, University of Salford, Salford M5 4WT, United Kingdom; ^3^ Present Address: School of Medicine, University of Nottingham, City Hospital, Nottingham NG5 1PB, United Kingdom

**Keywords:** Chromosome Section, spindle assembly checkpoint, taxol, APC/C-Cdc20, FBW7, Bcl-xL

## Abstract

Microtubule-binding drugs such as taxol are frontline treatments for a variety of cancers but exactly how they yield patient benefit is unclear. In cell culture, inhibiting microtubule dynamics prevents spindle assembly, leading to mitotic arrest followed by either apoptosis in mitosis or slippage, whereby a cell returns to interphase without dividing. Myeloid cell leukaemia-1 (Mcl-1), a pro-survival member of the Bcl-2 family central to the intrinsic apoptosis pathway, is degraded during a prolonged mitotic arrest and may therefore act as a mitotic death timer. Consistently, we show that blocking proteasome-mediated degradation inhibits taxol-induced mitotic apoptosis in a Mcl-1-dependent manner. However, this degradation does not require the activity of either APC/C-Cdc20, FBW7 or MULE, three separate E3 ubiquitin ligases implicated in targeting Mcl-1 for degradation. This therefore challenges the notion that Mcl-1 undergoes regulated degradation during mitosis. We also show that Mcl-1 is continuously synthesized during mitosis and that blocking protein synthesis accelerates taxol induced death-in-mitosis. Modulating Mcl-1 levels also influences slippage; overexpressing Mcl-1 extends the time from mitotic entry to mitotic exit in the presence of taxol, while inhibiting Mcl-1 accelerates it. We suggest that Mcl-1 competes with Cyclin B1 for binding to components of the proteolysis machinery, thereby slowing down the slow degradation of Cyclin B1 responsible for slippage. Thus, modulating Mcl-1 dynamics influences both death-in-mitosis and slippage. However, because mitotic degradation of Mcl-1 appears not to be under the control of an E3 ligase, we suggest that the notion of network crosstalk is used with caution.

## INTRODUCTION

Microtubule binding agents are used extensively to treat ovarian, breast, prostate and lung cancer, as well as various leukemias [1]. While these drugs have impressive clinical efficacy, exactly how they yield patient benefit is unclear [2, 3]. This is in part because inhibiting microtubule dynamics impacts multiple aspects of tumor biology [4, 5]. Interfering with microtubules becomes particularly acute during mitosis when the interphase microtubule array is disassembled in order to construct the spindle apparatus responsible for chromosome segregation and cell division [1]. Disrupting spindle assembly results in persistent activation of the spindle assembly checkpoint (SAC) leading to a prolonged mitotic arrest [6-8]. After a protracted mitosis, cells either undergo death in mitosis (DiM), or exit without completing cell division and return to interphase, a process known as slippage [9-11]. Following slippage, post-mitotic responses then induce cell cycle arrest, senescence or apoptosis [12]. In response to agents that perturb mitosis, failure to undergo DiM and/or failure to efficiently engage post-mitotic responses can lead to proliferation of cells with highly abnormal genomes [13, 14].

Whether a cell dies in mitosis or undergoes slippage is best explained by the competing-networks model [10]. This model posits that mitotic fate is dictated by two independent networks, one slowly generating a death signal, the other slowly degrading Cyclin B1, leading to slippage. During a protracted arrest, Cyclin B1 levels slowly fall due to incomplete penetrance of the SAC [9]. Meanwhile, a poorly defined cell death signal becomes stronger [15]. Both networks contain thresholds and the fate of the cell is dictated by which threshold is breached first. Our understanding of the mechanisms responsible for Cyclin B1 degradation are well advanced [16]. However, much less is known about how apoptosis is regulated during a mitotic arrest.

Death in mitosis involves the intrinsic apoptosis pathway, with multiple members of the Bcl-2 family subject to regulation by mitosis-specific controls [17]. For example, the BH3-only pro-apoptotic protein Bim is ubiquitinated by the anaphase promoting complex (APC/C), an E3 ubiquitin ligase that also targets Cyclin B1 for degradation [18]. Bid, another BH3-only pro-apoptotic protein, is phosphorylated by Cdk1 [19]. Pro-survival Bcl-xL is also phosphorylated by Cdk1, weakening its ability to bind and inhibit pro-apoptotic proteins Bax and Bak [20, 21]. Mcl-1 is phosphorylated and ubiquitinated in mitosis, and several protein kinases and E3 ubiquitin ligases have been implicated. Cdk1 phosphorylates Mcl-1 on serine 64 and threonine 92, and when expressed in U2OS cells, Mcl-1 T92A resists degradation and suppresses nocodazole-induced apoptosis [22, 23]. Mcl-1 binds the APC/C co-factor Cdc20 and has a putative D-box, an RXXL motif required for co-factor binding. Mutating the RXXL motif or inhibiting APC/C-Cdc20, either by depletion of Cdc20 or the APC subunit APC3, stabilizes Mcl-1 in mitotic-arrested U2OS cells [22]. These observations suggest a simple model whereby Mcl-1 is targeted for degradation during a mitotic arrest by Cdk1-dependent APC/C-Cdc20 ubiquitination. However, other mechanisms are also implicated. In interphase, Mcl-1 is targeted for degradation by FBW7, an F-box adapter involved in SCF-mediated ubiquitination [24, 25]. Inhibiting FBW7 also stabilizes Mcl-1 in mitosis and enhances slippage [26]. The E3 ligase first shown to be involved in Mcl-1 ubiquitination was MULE/HUWE1 [27]. Inhibiting MULE also stabilizes Mcl-1 in mitotic-arrested cells [28, 29]. Reconciling these different observations is difficult but may reflect redundancy, cell line variation and/or ‘belt and braces’ protection to ensure that an overly protracted mitosis sensitizes cells to undergo apoptosis [17].

Despite these complexities, because Mcl-1 is degraded during a mitotic arrest, it could act as the death timer evoked by the competing-networks model [10, 30, 31]. In its original formulation, the competing-networks model suggested that the two networks are independent [10]. Observations indicating that Mcl-1 interacts with both apoptotic and mitotic regulators, raises the possibility that there may however be crosstalk between the networks that define mitotic cell fate. To test this possibility, we analyzed the effect of Mcl-1 degradation on both death in mitosis and slippage.

## RESULTS

### Mcl-1 is synthesized and degraded in mitosis

RNAi-mediated inhibition of Mcl-1 can accelerate death in mitosis (DiM), at least in some cell lines [29, 32]. In addition, Mcl-1 is degraded during a mitotic arrest [22]. This suggests that Mcl-1 degradation acts as a death in mitosis timer. If this is the case, then blocking Mcl-1 degradation should delay DiM. To test this, we turned to RKO cells, a colon cancer line that typically undergoes DiM and rarely slips [10]. Consistent with previous reports, Mcl-1 levels fell when RKO cells were arrested in mitosis with taxol (Fig. S1A). Moreover, upon exposing taxol-arrested RKO cells to the proteasome inhibitor MG132, Mcl-1 levels increased (Fig. S1B). This indicates that not only is Mcl-1 degraded in mitosis, but that it is also synthesized during the arrest. Indeed, blocking protein synthesis with cycloheximide led to a rapid decline in Mcl-1 levels (Fig. S1C).

**Figure 1:**
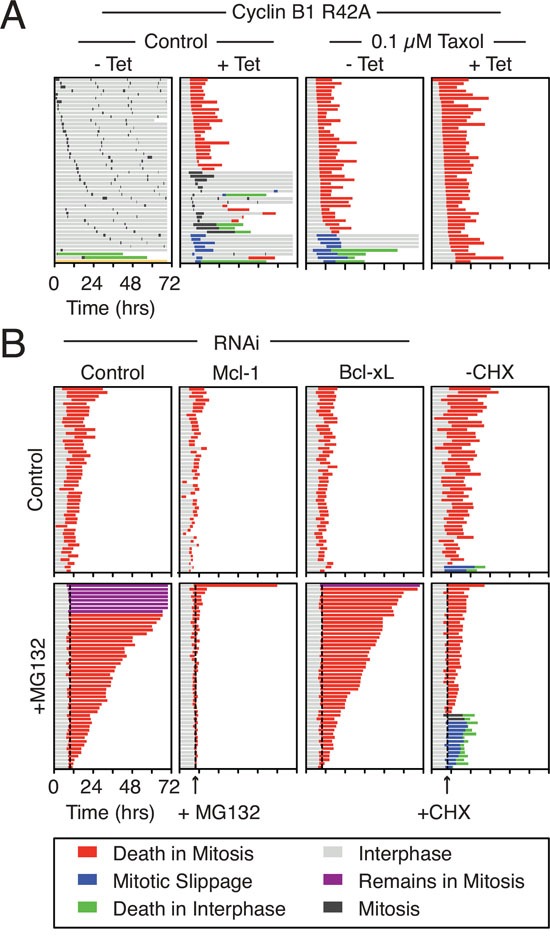
Mcl-1 is synthesized and degraded in mitosis **A.** Cell fate profiles of RKO Cyclin B1 R42A cells following treatment with 0.1 μM taxol and 1 μg/ml tetracycline for 72 hours. **B.** Cell fate profiles of RKO Cyclin B1 R42A cells following a 24-hour transfection with Mcl-1 and Bcl-xL siRNAs before treatment with taxol, tetracycline (1 μg/ml) and MG132 (20 μM) or cycloheximide (CHX) (30 μg/ml) at 10 hours. The dotted line shows when MG132 and cycloheximide were added. Only cells that had entered mitosis 2.5 hours before drug addition were analyzed.

Next, we set out to determine whether blocking Mcl-1 degradation with MG132 delayed death in mitosis. However, because MG132 also blocks the slow degradation of Cyclin B1 it abolishes slippage which complicates interpretation of the results [9]. Therefore to focus on Mcl-1's role in DiM, we created experimental conditions whereby slippage was prevented by expression of a stabilized version Cyclin B1, namely the R42A mutant [33]. Combining taxol with tet-induction of Cyclin B1 R42A completely blocked slippage: every cell we analyzed by time-lapse imaging underwent death in mitosis (Fig. 1A, Fig. S1D)

Addition of MG132 to taxol-treated Cyclin B1 R42A cells had a profound effect. Whereas control cells died after an average of 10.2 hours, MG132-treated cells took on average 31.2 hours to commit to DiM (Fig. 1B, left panel, Fig. S1E, S1F). To determine whether Mcl-1 was required for this prolonged survival, we used RNAi to inhibit Mcl-1. As an additional control we also performed Bcl-xL RNAi. Depletion of Mcl-1 and Bcl-xL levels accelerated DiM with cells taking on average 3.5 and 6.4 hours, respectively. Strikingly, Mcl-1 RNAi completely reversed the effect of MG132. By contrast, Bcl-xL RNAi had little effect. Thus, the simplest explanation for these observations is that the prolonged death delay induced by MG132 is due to inhibition of Mcl-1 degradation.

Consistent with continued synthesis of Mcl-1 maintaining survival during a mitotic arrest, addition of cycloheximide accelerated DiM (Fig. 1B, right panel). Interestingly, 26% of the cycloheximide-treated cells avoided DiM by undergoing slippage. Note that in the absence of taxol, the mitotic arrest induced by Cyclin B1 R42A is not fully penetrant (Fig. 1B, Fig. S1G); we therefore suspect that enhanced slippage in this case is caused by cycloheximide reducing mitotic synthesis of Cyclin B1, thereby accelerating Cyclin B1's decline even though DiM is also accelerated [34]. Taken together, these observations support the notion that Mcl-1 is a key mitosis survival factor and that degradation of Mcl-1 defines time to death during a protracted mitotic arrest.

### Analysis of E3 ligases implicated in mitotic degradation of Mcl-1

**Figure 2:**
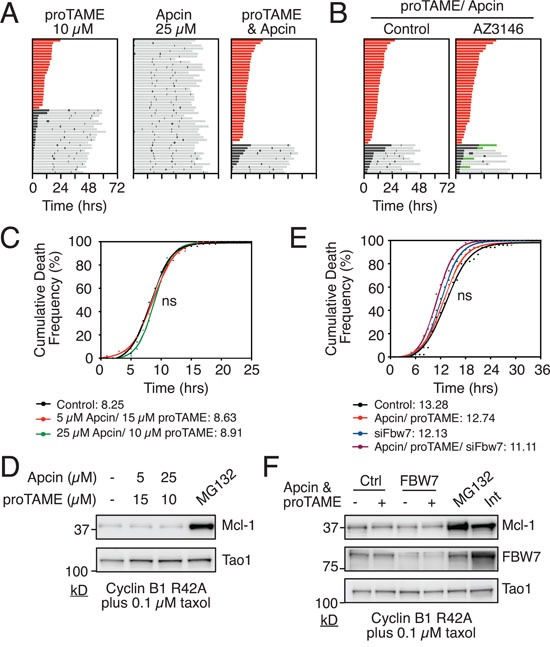
Analysis of E3 ligases implicated in mitotic degradation of Mcl-1 **A.** Cell fate profiles of RKO Cyclin B1 R42A cells treated with APC/C-Cdc20 inhibitors proTAME and/or Apcin for 72 hours using concentrations indicated. Zero hours represents mitotic entry. **B.** Cell fate profiles of RKO Cyclin B1 R42A cells co-treated with proTAME, Apcin and the Mps1 inhibitor AZ3146 for 72 hours. Zero hours represents mitotic entry. **C.** Cumulative death frequency of RKO Cyclin B1 R42A cells co-treated with taxol, tetracycline and proTAME/Apcin. Mann-Whitney U test, p > 0.05 **D.** Immunoblot of Mcl-1 levels in RKO Cyclin B1 R42A cells after 16 hours co-treatment with 0.1μM taxol, 1 μg/ml tetracycline and proTAME/Apcin at concentrations indicated. MG132 was added after 10 hours treatment. **E.** Cumulative death frequency graph of RKO Cyclin B1 R42A FBW7 RNAi cells exposed to proTAME/Apcin, 0.1μM taxol and 1 μg/ml tetracycline. Mann Whitney U test, ns p > 0.05. **F.** Immunoblot showing Mcl-1 and FBW7 levels in RKO Cyclin B1 R42A cells following transfection with FBW7 siRNA followed by exposure to proTAME/Apcin, 0.1μM taxol and 1 μg/ml tetracycline for 16 hours. Interphase sample was taken at zero hours.

Multiple E3 ligases are implicated in targeting Mcl-1 for degradation, including the APC/C-Cdc20 [35]. Because the APC/C-Cdc20 is responsible for slow degradation of Cyclin B1 during a prolonged mitotic arrest, it is a good candidate for mitotic degradation of Mcl-1. If this is the case, then blocking APC/C-Cdc20 should delay time to DiM in taxol-treated Cyclin B1 R42A cells. To test this we treated cells with two APC/C-Cdc20 inhibitors, namely pro-TAME and Apcin (Fig. S2) [36, 37]. In the absence of taxol and Cyclin B1 R42A, pro-TAME blocked mitotic progression (Fig. 2A), consistent with APC/C-Cdc20 inhibition. In isolation, Apcin had little effect but enforced the pro-TAME-mediated block, resulting in 78% of the population undergoing DiM. Importantly, this effect was not due to SAC activation as a consequence of cohesion fatigue [38] because the Mps1 inhibitor AZ3146 did not override the pro-TAME/Apcin-induced arrest (Fig. 2B).

Having established that combining pro-TAME and Apcin efficiently blocks APC/C-Cdc20 activity in RKO cells, we asked whether this would delay time to death in taxol-treated Cyclin B1 R42A cells. However, when we analyzed the cumulative death frequency, there was no difference between cells treated with or without the pro-TAME/Apcin combination (Fig. 2C). As DiM kinetics in this cell line are responsive to modulation of Mcl-1 levels, this suggests that while the APC/C-Cdc20 may contribute to Mcl-1 degradation during mitosis, it is not essential. Consistently, in contrast to MG132, the pro-TAME/Apcin combination did not stabilize Mcl-1 levels in mitosis (Fig. 2D). We therefore turned to another E3 ligase implicated in Mcl-1 degradation, the SCF complex. Inhibition of the SCF subunit FBW7 by RNAi, either alone or in combination with proTAME/Apcin, had no significant effect on Mcl-1 levels or the time to death in taxol-treated Cyclin B1 R42A cells (Fig. 2E, 2F). Moreover, RNAi-mediated inhibition of MULE, either alone or in combination with FBW7 RNAi and/or proTAME/Apcin, had no significant effect on DiM (Fig. S3). Thus, in the context of this model system, we do not have any compelling evidence linking mitotic Mcl-1 degradation to APC/C-Cdc20, FBW7 and MULE. This could suggest that the modalities used here are simply not penetrant enough to have a significant inhibitory effect on Mcl-1 degradation. Alternatively, it could be that other mechanisms contribute to Mcl-1 degradation even when APC/C-Cdc20, FBW7 and MULE are inhibited.

### Analysis of Mcl-1's putative D-box

**Figure 3:**
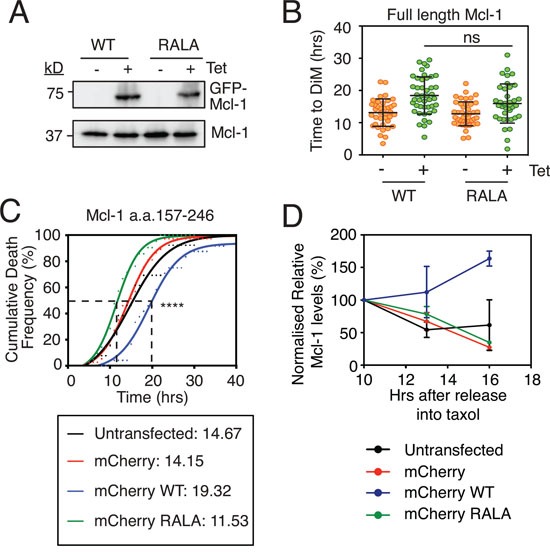
Analysis of Mcl-1's putative D-box **A.** Immunoblot of endogenous Mcl-1 (lower panel) and GFP-tagged Mcl1 (upper panel) following 24-hour 1 μg/ml tetracycline induction. **B.** Quantitation of time to mitotic death of RKO cells expressing Mcl-1^WT^ and Mcl-1^RALA^ following incubation with 0.1μM taxol and 1 μg/ml tetracycline. Zero hours represents mitotic entry. Mann Whitney U test, ns p > 0.05. **C.** Cumulative death frequency of RKO Cyclin B1 R42A cells transiently transfected with pLNCX2 plasmids expressing myc-tagged mCherry fused to Mcl-1 competitor fragments (a.a. 157-246) with or without the RALA mutation. Transfected cells were identified by fluorescent microscopy and tracked by phase contrast microscopy. Mann Whitney U test between mCherry WT and mCherry RALA, **** p < 0.0001. **D.** Quantitation of two independent immunoblots showing relative Mcl-1 levels in RKO Cyclin B1 R42A cells transiently transfected with the mCherry-Mcl-1 WT and RALA fragments exposed to 0.1 μM taxol for the times indicated.

Because inhibiting APC/C-Cdc20 and FBW7 had little effect on either Mcl-1 degradation or time to death, we turned to a more direct approach, namely mutating potential degrons in Mcl-1. In particular, we focused on a potential D-box. Note that D-boxes contain RXXL motifs and are typically found in APC/C-Cdc20 substrates [16]. Importantly, mutating an RXXL motif in Mcl-1 was previously shown to suppress its degradation in mitosis [22]. We therefore generated tet-inducible RKO cells expressing GFP-tagged wild type Mcl-1 and a mutant, Mcl-1^RALA^, where arginine 207 and leucine 210 in the RXXL motif are mutated to alanine (Fig. 3A). Consistent with Mcl-1's pro-survival effect, tet-induction of the wild type protein delayed time to death in taxol from 13.1 hours to 18.4 hours (Fig. 3B). If the Mcl-1^RALA^ mutant resisted mitotic degradation, then we expected that it might have a more penetrant effect on DiM. However, while Mcl-1^RALA^ also extended time to death in taxol, the effect was comparable to the wild type (Fig. 3B).

Immunoblotting suggested that the ectopic Mcl-1 proteins were expressed at similar levels to the endogenous (Fig. 3A). We therefore asked whether forced overexpression might reveal a difference between the wild type and the Mcl-1^RALA^ mutant. Moreover, we wanted to test the Mcl-1^RALA^ mutant in cells expressing Cyclin B1 R42A. Therefore, we used transient transfections of RKO cells to force overexpression. Also, rather than expressing full length Mcl-1, we expressed a 90 amino acid fragment (a.a. 157-246) encompassing the putative D-box. We reasoned that if this fragment could compete with the endogenous protein for APC/C-Cdc20 binding, this might delay degradation of endogenous Mcl-1 and thus delay DiM. By contrast, if the RALA mutant was deficient for APC/C-Cdc20 binding, then it should have no effect on DiM. Note that this approach is conceptually similar to the experiment that laid the foundation for the discovery of Securin whereby addition of a fragment of Cyclin B1 containing the D-box to *Xenopus* egg extracts competed endogenous Cyclin and Securin away from APC/C-Cdc20, thereby inhibiting mitotic exit and anaphase onset [39]. To test this, RKO Cyclin B1 R42A cells were transiently transfected with constructs expressing mCherry-tagged Mcl-1 fragments, treated with taxol and cells entering mitosis were analyzed by time-lapse microscopy. Analysis of the cumulative death frequency in control cells or those transfected with mCherry alone showed that the time taken for 50% of the cells to undergo DiM (i.e. the T_50_) was 14.2 hours (Fig. 3C). Overexpressing the Mcl-1 fragment extended the T_50_ to 19.3 hours. Strikingly, this was reversed by mutating the RXXL motif; analysis of the RALA fragment showed that the T_50_ was reduced to 11.5 hours. One explanation for these observations is that endogenous Mcl-1 interacts with APC/C-Cdc20 and that overexpressing the D-box fragment, but not the Mcl-1^RALA^ fragment, acts as a competitor thus inhibiting Mcl-1 degradation and thereby extending time to death. Consistently, expression of the D-box fragment, but not the Mcl-1^RALA^ fragment, stabilized endogenous Mcl-1 during a taxol-mediated arrest (Fig. 3D).

Taken together, these observations suggest that while Mcl-1 may indeed engage with APC/C-Cdc20, the significance is unclear. In particular, while overexpressing the RXXL fragment supports an interaction between Mcl-1 and APC/C-Cdc20, the experiments analyzing full length Mcl-1 expressed at more physiological levels do not support the notion. Moreover, as described above, in our hands, inhibiting APC/C-Cdc20 had no obvious affect on Mcl-1 degradation or time to death.

### Examination of a lysine-less Mcl1 mutant

**Figure 4:**
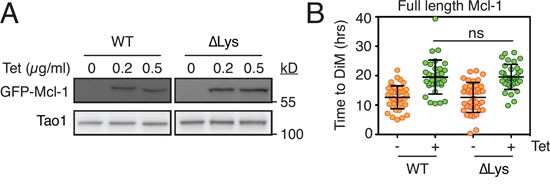
Analysis of a lysine-less Mcl1 **A.** Immunoblots showing tetracycline-mediated induction of GFP-tagged wild type mouse Mcl-1 (WT) and a lysine-less mutant (ΔLys) whereby all 14 lysine residues are mutated to arginine. **B.** Quantitation showing time to death-in-mitosis for RKO cells expressing wild type Mcl-1 and the lysine-less mutant following exposure to 0.1 μM taxol. Transgenes were induced with 0.5 μg/ml tetracycline.

In our hands, inhibiting three E3 ligases previously implicated in mitotic Mcl-1 degradation had no obvious effect on Mcl-1 levels or the time to DiM. If this was due to redundancy and/or the involvement of additional E3 enzymes, we predicted that a lysine-less Mcl-1 should be non-degradable and therefore resist mitotic proteolysis and thus extend time to death in taxol-treated cultures. To test this, we generated stable tet-inducible RKO cell lines expressing either wild type Mcl-1 or a lysine-less mutant [40] whereby all 14 lysines are mutated to arginine (Mcl-1 ΔLys, Fig. 4A). Significantly, while both wild type and the lysine-less mutant delayed the time to DiM, they did so to the same extent (Fig. 4B). This suggests that mitotic degradation of Mcl-1 during mitosis may not be mediated by the canonical ubiquitin-proteasome pathway. Note that previously, Stewart et al. showed that the lysine-less Mcl-1 is also degraded in interphase cells in a manner comparable to the wild type protein [40].

### Mcl-1 levels influence slippage

**Figure 5:**
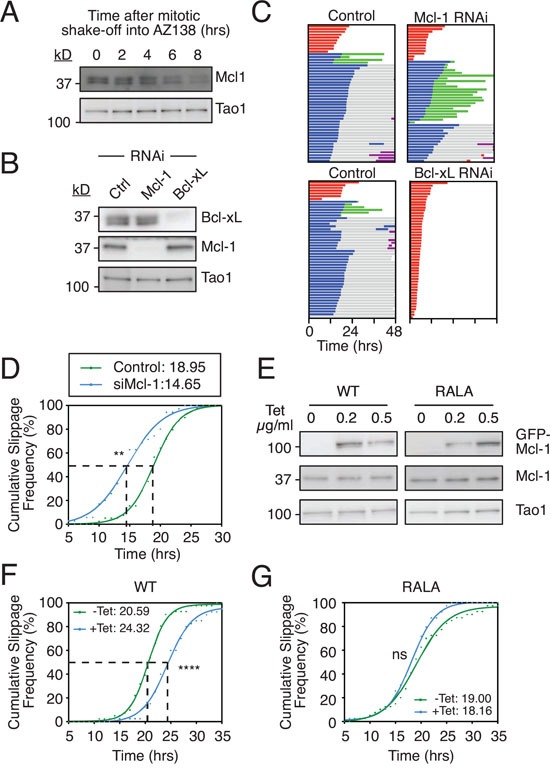
Mcl-1 levels influences slippage **A.** Immunoblot showing Mcl-1 levels in DLD-1 cells arrested in mitosis. Cells were treated with AZ138 for 4 hours before mitotic shake-off into AZ138, then harvested at the times indicated. **B.** Immunoblot showing Mcl-1 and Bcl-xL levels following RNAi. **C.** Cell fate profiles of DLD-1 cells treated with AZ138 following Mcl-1 or Bcl-xL RNAi. Zero hours represents mitotic entry. **D.** Cumulative slippage frequency of DLD-1 cells treated with AZ138 following Mcl-1 RNAi. Mann Whitney U test, ** p < 0.01. **E.** Immunoblot of endogenous Mcl-1 and GFP-tagged Mcl-1 in DLD-1 cells following tetracycline induction. **F** and **G.** Cumulative slippage frequency of DLD-1 Mcl-1^WT^ and Mcl-1^RALA^ cells following induction with 0.5 μg/ml tetracycline and AZ138 exposure. Mann Whitney U test, **** p < 0.0001, ns p > 0.05.

Overexpressing the Mcl-1 fragment containing the putative D-box suggests that while Mcl-1 may not be a *bona fide* E3 ligase substrate, it can engage APC/C-Cdc20 during a prolonged mitotic arrest. While the significance of this in the context of regulating DiM is unclear, we reasoned that if it was D-box-dependent it might influence the interaction between Cyclin B1 and APC/C-Cdc20, and thus influence slippage. To test this, we turned to DLD-1 cells, another colon cancer cell line that tends to undergo slippage following a prolonged arrest [10]. Note that as in RKO cells, Mcl-1 protein levels decline in DLD-1 cells arrested in mitosis (Fig. 5A). First, we inhibited Mcl-1 by RNAi (Fig. 5B) then added AZ138, an Eg5 kinesin inhibitor. In the control population, 80% of cells underwent slippage (Fig. 5C). Of these, 10% then died in the following interphase. Upon RNAi-mediated inhibition of Mcl-1, a similar proportion (74%) underwent slippage, but of these 62% then died in interphase, demonstrating that Mcl-1 is an important post-mitotic survival factor, at least in this context. Strikingly, RNAi-mediated inhibition of Bcl-xL shifted the fate profile from slippage to DiM, indicating that Bcl-xL is an essential mitotic survival factor in DLD-1 cells. By contrast, note that in RKO cells, Mcl-1 compensates for Bcl-xL loss during a prolonged arrest (Fig. S1F and see also [32]). Interestingly, comparing RKO and DLD-1 with four additional cell lines showed a provocative correlation between relative Mcl-1 levels and slippage. Specifically, both DLD-1 and HCT116, which are slippage prone in the presence of taxol (Fig. S4A), have relatively low levels of Mcl-1 (Fig. S4B) and are resistant to Mcl-1 RNAi (Fig. S5). By contrast, RKO, HeLa, HT29 and H1703, which more are prone to death in mitosis (Fig. S4A), have relatively higher levels of Mcl-1 (Fig. S4B).

Because Mcl-1-deficient DLD-1 cells still underwent slippage, we measured the time from mitotic entry to exit to determine if slippage kinetics were affected. Analysis of the cumulative slippage frequency showed that the time taken for 50% of the control cells to slip (i.e. the T_50_) was 19.0 hours. Mcl-1 RNAi reduced the T_50_ to 14.7 hours (Fig. 5D). Thus, suppressing Mcl-1 levels accelerates slippage. We therefore asked whether artificially elevating Mcl-1 levels would have the opposite effect, i.e. delay slippage. To test this we generated tet-inducible DLD-1 lines expressing either wild type Mcl-1 or the Mcl-1^RALA^ mutant (Fig 5E). Induction of the wild type protein delayed slippage by 3.7 hours (Fig 5F). By contrast the Mcl-1^RALA^ mutant had no impact on the T_50_ (Fig. 5G). Thus, artificially elevating Mcl-1 levels does indeed delay slippage and this effect appears to require the putative D-box. One possible explanation for these observations is that Mcl-1 competes with Cyclin B1 for APC/C-Cdc20 binding in a D-box dependent manner such that when Mcl-1 levels are reduced, Cyclin B1 is degraded faster thereby accelerating slippage. Conversely when Mcl-1 is overexpressed, Cyclin B1 degradation is inhibited, thereby delaying slippage.

### Suppressing Mcl-1 rescues delayed slippage induced by Bax/Bak depletion

**Figure 6:**
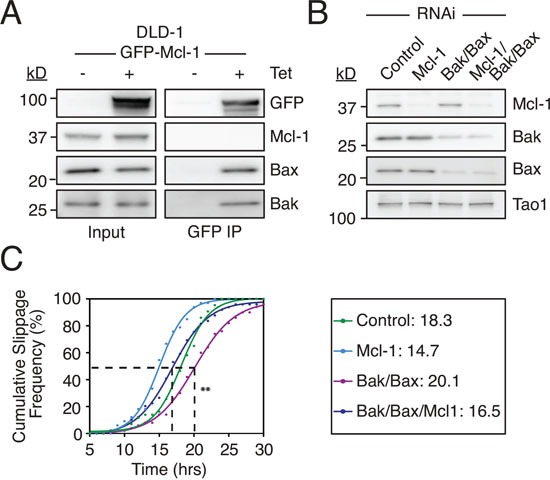
Suppressing Mcl-1 rescues delayed slippage induced by Bax/Bak depletion **A.** Immunoblot of Bak, Bax and Mcl-1 levels in DLD1 Mcl-1^WT^ cells following immunoprecipitation of GFP-AID-Mcl-1 induced with 0.5 μg/ml tetracycline. **B.** Immunoblot of Mcl-1, Bak and Bax levels 24 hours after transfection of the indicated siRNAs. **C.** Cumulative slippage frequency of DLD-1 cells treated with AZ138 following RNAi-mediated inhibition of Bak, Bax and Mcl-1. Mann Whitney U test, ** p < 0.01.

The ability of Mcl-1 to influence slippage as shown above could help explain a recent and surprising observation, namely that that RNAi-mediated inhibition of Bax and Bak delayed slippage [41]. Because Mcl-1 binds Bax and Bak (Fig. 6A and see also [42]), their removal by RNAi would be expected to liberate Mcl-1, thereby enabling it to engage with the APC/C-Cdc20 and/or the proteasome and thus delay Cyclin B1 degradation. To test this we depleted Bax and Bak in DLD-1 cells and determined the cumulative slippage frequency (Fig. 6B). Consistent with the recent report, Bax/Bak co-RNAi delayed the T_50_ for time to slippage from 18.3 hours to 21.1 hours, indicating delayed slippage (Fig. 6C). Consistent with our data shown above, Mcl-1 RNAi reduced the T_50_ to 14.7 hours, indicating accelerated slippage. Importantly, depleting Mcl-1 in Bax/Bak RNAi cells restored the time to slippage, with a T_50_ of 16.5 hours. Moreover, degradation of an mCherry D-box fusion [10] was accelerated following Mcl-1 RNAi, and this was partially rescued by co-depletion of Bax and Bak (Fig. S6). Thus, a simple explanation for the delayed slippage observed in Bax/Bak-deficient cells is the ability of liberated Mcl-1 to compete with Cyclin B1 and thus slow degradation of the latter.

### Elevating Mcl-1 levels delays both death in mitosis and slippage

**Figure 7:**
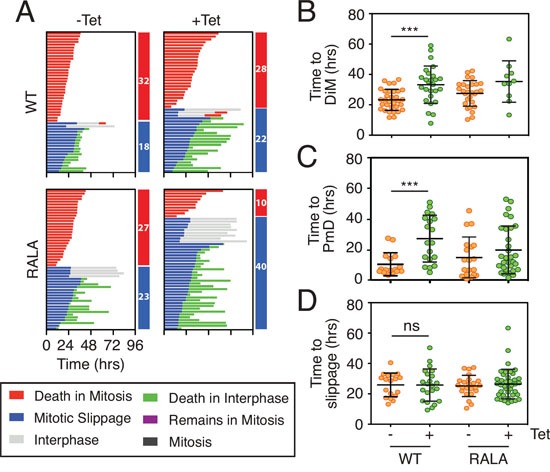
Overexpressing Mcl-1 levels delays both death in mitosis and slippage **A.** Cell fate profiles and **B, C, D** quantification of time in mitosis for cells committed to death in mitosis (DiM), post-mitotic death or slippage in DLD-1 Mcl-1^WT^ and DLD-1 Mcl-1^RALA^ cell lines treated with 0.5 μg/ml tetracycline and 6.6 μM nocodazole. Zero hours represents mitotic entry. Mann Whitney U test, *** p < 0.001, ns p > 0.05.

We previously showed that there is extensive inter-line variation in terms of how different cancer cell lines respond to a protracted mitotic arrest [10]. By using RKO and DLD-1 cells for the experiments described above, we exploited this inter-line variation to focus on DiM and slippage respectively; whereas RKO typically undergo DiM, DLD-1 are slippage prone. Our observations show that overexpressing Mcl-1 can delay DiM in RKO and delay slippage in DLD-1. In principle therefore, overexpressing Mcl-1 should delay DiM as well as slippage in DLD-1 cells. To test this, we re-tuned the DLD-1 experimental system to favor DiM instead of slippage. Rather than using an Eg5 inhibitor, we used a high dose of nocodazole; under these conditions, 64% of the DLD-1 cells underwent DiM, presumably due to super-activation of the SAC and more penetrant APC/C-Cdc20 inhibition (Fig. 7A). Tet-induction of wild type Mcl-1 had multiple effects. Firstly, of the cells that underwent DiM, time to death was delayed from 23.2 hours to 33.2 hours (Fig. 7B). Secondly, of the cells that underwent slippage, post-mitotic death was delayed from 10.3 hours to 27.1 hours (Fig. 7C). Overexpressing the Mcl-1^RALA^ mutant also delayed DiM from 27.5 hours to 35.4 hours (Fig. 7B).

The competing networks model predicts that delaying DiM provides more time for Cyclin B1 degradation and should therefore shift the fate profile from DiM to slippage. Interestingly, overexpressing Mcl-1 only had a marginal effect on the proportion of cells undergoing slippage, from 36% in controls to 44% (Fig. 7A). This modest effect could be because overexpressing Mcl-1 not only inhibits DiM but also slows Cyclin B1 degradation by engaging with the APC/C-Cdc20. Consistent with the Mcl-1^RALA^ mutant not engaging APC/C-Cdc20, overexpression of Mcl-1^RALA^ had a substantial effect on the number of cells undergoing slippage, increasing it from 48% in controls to 80% (Fig. 7A). Moreover, time to slippage was unaffected in DLD-1 cells expressing either wild type Mcl-1 or the RALA mutant (Fig. 7D). Thus, taken together, these results show that overexpressing Mcl-1 in DLD-1 cells delays both DiM and slippage, and that while the Mcl-1^RALA^ mutant is as effective at delaying DiM, it is less effective at delaying slippage. Although we have no direct evidence that wild type Mcl-1 binds Cdc20, these observations are consistent with the notion put forward by Harley et al., namely that Mcl-1 engages the APC/C-Cdc20 in a D-box dependent manner [22].

### Delaying slippage enhances post-mitotic apoptosis

**Figure 8:**
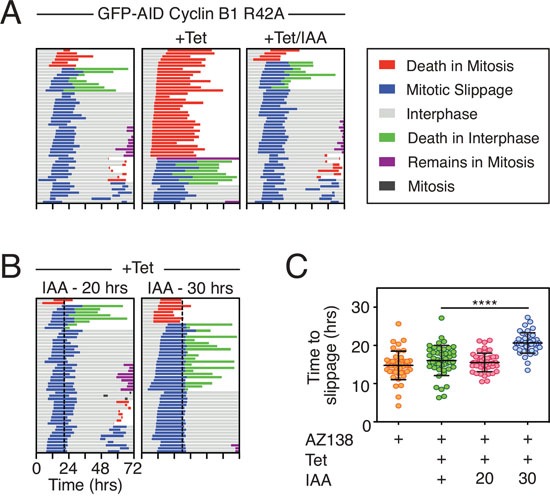
Delaying slippage enhances post-mitotic apoptosis **A, B.** Cell fate profiles of DLD-1 GFP-AID-Cyclin B1 R42A cells treated with 1 μM AZ138, 1 μg/ml tetracycline and 500 μM IAA. In panel (B) dotted lines show addition of IAA after 20 and 30 hours. **C.** Quantification of time to slippage. Mann Whitney U test, **** p < 0.0001.

As noted above, overexpressing Mcl-1 in DLD-1 cells delayed post-mitotic apoptosis (Fig. 7A). Also, Mcl-1 RNAi increased the proportion of cells undergoing post-mitotic apoptosis following slippage from 10% to 62% (Fig. 5C). In addition, it was previously shown that inhibition of Mcl-1 suppresses cell cycle progression in interphase following nocodazole treatment [43]. This points at a post-mitotic survival role for Mcl-1. We therefore asked whether mitotic degradation of Mcl-1 might also act as a post-mitotic death timer. If so, then the longer a cell spends arrested in mitosis, the more likely Mcl-1 levels are to fall such that even if it survives for long enough to undergo slippage, the more likely it is to die in interphase. If correct, one might expect there to be an inverse correlation between the time spent arrested in mitosis before slippage and the time from exit to death. Indeed, in some experimental conditions this does appear to be the case (Fig. S7). However, in other conditions there was no obvious correlation. We therefore set out to test more directly whether a longer mitotic arrest prior to slippage was more likely to induce post-mitotic death. To do this, we obtained a DLD-1 cell line constitutively expressing a myc-tagged OsTir transgene and generated derivatives expressing a tet-inducible stabilized Cyclin B1 R42A mutant fused to an AID tag so that it could be degraded at will by addition of IAA (Fig. S8A and see also [44, 45]). Quantitating GFP fluorescence indicated that 50% of the GFP-AID-Cyclin B1 R42A protein was degraded within 22 minutes following IAA treatment (Fig. S8B). When an uninduced population was exposed to the Eg5 inhibitor AZ138, 88% of cells arrested in mitosis and underwent slippage after an average of 14.8 hours (Fig. 8A). Upon tet-induction of GFP-AID-Cyclin B1 R42A, 70% of the AZ138-treated cells underwent DiM with a mean arrest time of 29.6 hours. Addition of IAA at the same time as the tetracycline reverted this phenotype, consistent with IAA targeting the GFP-AID-Cyclin B1 R42A for SCF-Tir1-mediated degradation. Next, we tet-induced GFP-AID-Cyclin B1 R42A, exposed the cells to AZ138 then added IAA either 20 or 30 hours later (Fig. 8B). When the IAA was added after 20 hours, it took on average 6.9 hours for the cells to exit mitosis, presumably because despite degrading the AID-tagged exogenous Cyclin B1 R42A, there was sufficient endogenous Cyclin B1 to maintain the mitotic state for several hours. Indeed, when we added the IAA after 30 hours, it took only 2.4 hours on average to trigger mitotic exit, consistent with further depletion of the endogenous protein; i.e. prior to addition of the IAA, the mitotic state was dependent on the exogenous GFP-AID-Cyclin B1 R42A. Moreover, prolonging the addition of IAA to 30 hours significantly extended the time to slippage (Fig. 8C). When the IAA was added after 20 hours, only 16% of the cells underwent post-mitotic death (Fig. 8B). By contrast, adding the IAA after 30 hours resulted in 42% of the cells undergoing post-mitotic death. Thus, adding IAA after 30 hours, allowed us to keep the cells in mitosis for long enough to deplete the endogenous Cyclin B1 but then drive them out before they underwent DiM. Under these conditions, cells were now much more likely to undergo post-mitotic death. One explanation to account for this is that extending the mitotic arrest provided more time for Mcl-1 degradation such that pro-survival function in the next interphase was compromised. However, it is also possible that extending the mitotic arrest provided more time for damage accumulation, regardless of Mcl-1 degradation [43, 46, 47]. Consistent with this latter notion, we noted that when the IAA was added after 20 hours, 60% of the cells entered a second mitosis. By contrast, adding the IAA after 30 hours resulted in only 7% of the cells entering a second mitosis. Nevertheless, these data strongly support the notion that the longer the duration of the mitotic arrest, the more likely it is that post-mitotic responses will be activated.

## DISCUSSION

We set out to define Mcl-1's role in mitotic cell fate determination. Initially, we focused on the role Mcl-1 degradation plays in determining the onset of apoptosis during a prolonged mitotic arrest. Our observations confirm that Mcl-1 is an important mitotic survival factor: overexpressing Mcl-1 delays DiM and RNAi-mediated inhibition accelerates DiM. Our observations also confirm that Mcl-1 levels decline during a prolonged mitotic arrest. This decline is reduced when the proteasome is inhibited and accelerated when protein synthesis is suppressed. Importantly, when slippage is completely blocked, i.e. in taxol-treated cells expressing a stabilized form of Cyclin B1, inhibiting the proteasome has a strikingly penetrant effect, delaying DiM by many hours. Conversely, blocking protein synthesis accelerates DiM. Taken together, these observations indicate that Mcl-1 is continuously synthesized and degraded during a mitotic arrest, and that mitotic degradation of Mcl-1 could serve as the death timer evoked by the competing-networks model.

Canonical proteasome-mediated protein degradation depends on the ubiquitin system, and three E3 ubiquitin ligases have been implicated in targeting Mcl-1 for degradation during mitosis [22, 26, 29]. However, we have not been able to generate compelling evidence that APC/C-Cdc20, FBW7 or MULE are involved. Moreover, we have no compelling evidence that the activity of these ligases is required for DiM. This could reflect redundancy; however, simultaneously inhibiting all three E3 ligases had no obvious effect. Consistently, Shi et al. reported only a mild effect upon co-inhibition of MULE and Cdc20 [29]. While we cannot rule out the possibility that the modalities employed here are simply not penetrant enough to block Mcl-1 ubiquitination, our observations raise the possibility that Mcl-1 is not a *bona fide* target of these E3 ligases during mitosis. Indeed, exogenous Mcl-1 harboring a mutation in the putative D-box did not prolong survival over and above that of a wild type transgene. One possibility is that during mitosis, Mcl-1 is degraded in a proteasome-dependent manner that does not require ubiquitination. This notion is supported by an analysis of Mcl-1 in mouse embryo fibroblasts [40]. Stewart et al. showed that Mcl-1 is degraded during interphase in a proteasome-dependent manner. However, an Mcl-1 mutant lacking all the lysine residues required for ubiquitination was degraded as efficiently as the wild type protein. Moreover, inhibiting the E1 ubiquitin-activating enzymes UAE and UBA6 had no effect on Mcl-1 degradation. And finally, an unmodified Mcl-1 substrate generated by *in vitro* transcription and translation was degraded by the 20S proteasome. Consistently, in our hands, the Mcl-1 mutant lacking lysine residues had no additional effect over and above the wild type control. Thus, although Mcl-1 may be ubiquitinated by multiple E3 ligases, it appears to be one of a growing list of proteins that can be degraded by the proteasome in an ubiquitin-independent manner [48]. This in turn provides a simple explanation for our observation showing that MG132 inhibits DiM in a Mcl-1-dependent manner but does not require APC/C-Cdc20, FBW7 or MULE.

The competing networks model, in its original formulation, proposed that the two networks responsible for mitotic cell fate are independent [10], a notion supported by predictive modeling [15]. However, a number of molecular interactions connect the mitotic and apoptotic pathways. For example, the master mitotic kinase Cdk-1 phosphorylates several apoptotic proteins including Caspase 9, Bcl-xL and Mcl-1 [21, 22, 49, 50]. In addition, Bim is targeted for degradation by APC/C-Cdc20 [18]. These molecular interactions raise the possibility that there is crosstalk between the two networks. Indeed, it was recently shown that inhibiting the SAC silencer p31^comet^ accelerated DiM, and that co-depletion of pro-apoptotic Bax and Bak delayed slippage [41]. The possibility of crosstalk presents a technical challenge as experimental modulation of one network may influence the other, thereby complicating interpretations. Indeed, SAC genes frequently manifest in anti-mitotic siRNA survival screens [32, 41, 51, 52], not because they directly promote apoptosis but because they are required to prevent slippage [53], which in turn allows time for the accumulation of death signals [10].

To focus on the two networks separately, here we first focused on RKO cells which typically undergo DiM [10]. Importantly, expressing a non-degradable Cyclin B1 in taxol-treated RKO cells completely blocked slippage, allowing us to use time-to-death to measure the integrity of the apoptotic network. Conversely, to focus on slippage we treated DLD-1 cells with an Eg5 inhibitor to create conditions which favor slippage [10]. These approaches demonstrate that experimentally altering Mcl-1 levels can influence both DiM and slippage, which on face value, appears to further support the notion of crosstalk. However, as discussed above, because degradation of Mcl1 in mitosis may be independent of an E3 ligase, it may not be a regulated phenomenon. Thus, the ability of Mcl-1 to influence slippage may be an epiphenomenon that arises during a prolonged mitotic arrest due to the presence of an RXXL motif that serves as a weak D-box allowing it to engage APC/C-Cdc20 in a non-regulated manner. If correct, the term crosstalk may be an over interpretation; we therefore suggest that in this case, network interference might be a more appropriate description.

The ability of Mcl-1 to be degraded by the proteasome independently of ubiquitination [40] may also provide alternative explanations for other observations that evoke crosstalk. For example, p31^comet^ RNAi inhibits SAC silencing and super-inhibits APC/C-Cdc20 thus delaying slippage by further inhibiting proteasome-dependent degradation of Cyclin B1 [54, 55]. This may alleviate competition between Cyclin B1 and Mcl-1 for proteasome binding, thereby accelerating Mcl-1 degradation and advancing the onset of DiM. Conversely, by co-depleting Bax and Bak, the level of free Mcl-1 is expected to increase [56], which could then increase competition with Cyclin B1 thus slowing degradation of the latter and delaying slippage. Further evidence that there may not be genuine crosstalk comes from the recent discovery that the oncogenic transcription factor MYC is a major determinant of mitotic cell fate [32]. Specifically, MYC promotes the death-in-mitosis network by upregulating a cluster of redundant, pro-apoptotic BH3-only proteins, namely Bim, Bid and Noxa, and downregulating pro-survival Bcl-xL. Importantly, while inhibiting MYC in RKO cells markedly delays DiM, it does not influence slippage [32]. Consequently, although inhibiting MYC promotes cell survival, the average amount of time spent arrested in mitosis increases from 17.1 to 21.3 hours. This supports the notion that the two networks responsible for mitotic cell fate are indeed independent. Moreover, by evoking the concept of network interference, our observations highlight the need for assays that can experimentally focus on DiM without interfering with slippage and vice versa.

## EXPERIMENTAL PROCEDURES

### Cell culture and drug treatments

Flp-In™ T-Rex™ DLD-1 and RKO cell lines were cultured as described [57]. HeLa, HCT116, HT-29 and H1703 were as described [10]. Flp-In™ T-Rex™ DLD-1 cells expressing TIR1-9myc were kindly provided by Andrew Holland [44]. Tet-inducible isogenic stable lines were generated as described [58, 59]. In brief, pcDNA5/FRT/TO-based plasmids containing tagged human and mouse Mcl-1 and Cyclin B1 cDNAs were co-transfected with pOG44 into Flp-In™ T-Rex™ cell lines using Lipofectamine Plus™. Transfected cells were selected using 80 μg/ml hygromycin (Sigma) and 8 μg/ml blasticidin then colonies pooled. Small molecule inhibitors dissolved in DMSO were used at the following concentrations: taxol (0.1 μM; Sigma), AZ138 (1 μM; [10]), AZ127 (2 μM; Tocris), MG132 (20 μM; Calbiochem), cycloheximide (30 μg/ml; Sigma), 3-Indoleacetic acid (IAA, 500 μM; Sigma). Transgenes were induced with addition of 0.25-1 μg/ml tetracycline (Sigma). Cell synchronization in early S-phase was performed with 2 mM thymidine (Sigma).

### Expression constructs

The human Mcl-1 open reading frame (ORF) was generated by RT-PCR using the Superscript^®^ One-Step System (Life Technologies) with total RNA isolated from RKO cells and the following primers: Forward 5′-ATGTTTGGCCTCAAAAGAAACGC-3′, reverse 5′-GGTCTTATTAGATATGCC-3′. Site directed mutagenesis of the RXXL motif in Mcl-1 was performed using the Q5^®^ Site-Directed mutagenesis kit following the manufacturers instructions with the following primers: 5′-TGGGGCCACCAGCGCGAAGGCGGCGG AGACCTTACGA-3′, 5′-TCGTAAGG TCTCCGCCGC CTTCGCGCTGGTGGCCCCA-3′. Standard molecular cloning techniques were used to insert the Mcl-1 ORF into pcDNA5/TO/GFP and pcDNA5/TO/GFP-AID vectors such that tags were fused to the C-terminus, placing the tagged ORFs downstream of a tet-regulatable CMV promoter. mCherry-Mcl-1 fragments encoding amino acids 157-246 were PCR amplified using the following primers: 5′-GACGGGTCACTACCCTCGACGCCG-3′, 5′-CAACGATTTCACATCGTCTTCGTT-3′ and cloned into a pLNCX2-based vector containing an N-terminal myc-tag. Mouse Mcl-1 ORFs were PCR amplified using p3XFlag-CMV10-Flag vectors (Addgene ID 32978 and 32979) and the following primers: 5′-TTTGGCCTGCGGAGAAACGCG-3′, 5′-CTATCTTATTAGATATGCCAG-3′ and cloned into pcDNA5/FRT/TO.

### RNAi and Transient transfections

For siRNA transfections, cells were seeded in a microclear 96 well plate (Greiner Bio-one) containing DharmaFECT 1 transfection reagent (Dharmacon), Opti-MEM (Life Technologies) and ON-TARGETplus SMARTpool siRNAs at a final concentration of 66 nM (Dharmacon). Sequences of siRNAs were as follows: Mcl-1 (5′-CGAAGGAAGUAUCGAAUUU-3′, 5′-GAUUAUCUCUCGGUACCUU-3′, 5′-GAAGGUGG CAUCAGGAAUG-3′, 5′-GGUUUGGCAUAUCUAAUA A-3′); Bcl-xL (5′-GGACAGCAUAUCAGAGCUU-3′, 5′-GAAAUGACCAGACACUGAC-3′, 5′-CCUACAAG CUUUCCCAGAA-3′, 5′-UUAGUGAUGUGGAAGAG AA-3′); Bak (5′-CGACAUCAACCGACGCUAU-3′, 5′-UAUGAGUACUUCACCAAGA-3′, 5′-GACGGCA GCUCGCCAUCAU-3′, 5′-AAUCAUGACUCCCAAGG GU-3′); Bax (5′-UGGGCUGGAUCCAAGACCA-3′, 5′-CUGAGCAGAUCAUGAAGAC-3′, ACAUGUUU UCUGACGGCAA-3′, 5-GUGCCGGAACUGAUCAG AA-3′); FBW7 (5′-GGGCACCAGUCGUUAACAA-3′, 5′-GUGAGUGGAUCUCUUGAUA-3′, 5′-GGAGUUG UGUGGCGGAUCA-3′, 5′-CAACAACGACGCCGAAU UA-3′); Non-targeting (5′-UGGUUUACAUGUCGA CUAA-3′, 5′-UGGUUUACAUGUUGUGUGA-3′, 5′-U GGUUUACAUGUUUUCUGA-3′, 5′-UGGUUUACAUG UUUUCCUA-3′). For transient transfections of plasmids, cells were plated 24 hours prior to transfection then transfections performed in antibiotic-free media with 0.5 μg DNA and DharmaFECT reagent (Dharmacon). Following transfections, cells were incubated at 37°C for 24 hours before addition of anti-mitotic agents. For synchronization, thymidine was added to cells during the transfection procedure.

### Immunoblotting

For immunoblotting, cells were seeded in 24 or 96 well plates (Corning) then later harvested by trypsinisation, centrifuged, washed and lysed in SDS buffer (0.35 M Tris pH 6.8, 0.1 g/ml sodium dodecyl sulfate, 93 mg/ml dithiothreitol, 30% glycerol, 50 μg/ml bromophenol blue). Proteins were resolved by SDS-PAGE then electro-blotted onto Immobilion-P membranes (Millipore). Membranes were blocked in 5% dried milk in TBST (50 mM Tris, pH 7.6, 150 mM NaCl, 0.1% Tween-20) then incubated overnight at 4°C with the following primary antibodies diluted in milk: rabbit anti-Mcl-1 (Santa-Cruz), sheep anti-Tao1 [60], mouse anti-Cyclin B1 (Millipore), rabbit anti-Bcl-xL (Cell Signalling), sheep anti-Bub3 (Holland and Taylor, unpublished), rabbit anti-FBW7 (Bethyl), mouse anti-Bak (Calbiochem), rabbit anti-Bax (Santa-Cruz), mouse anti-myc tag (4A6, Millipore), rabbit anti-GFP (Cell Signalling). Following TBST washes, blots were incubated with appropriate horseradish-peroxidase-conjugated secondary antibodies (Zymed). Bound secondaries were then detected by addition of EZ-ECL Chemiluminescence Reagent (Biological Industries) or Luminata™ Forte Western HRP Substrate (Millipore) and imaged using a Biospectrum 500 imaging system (UVP).

### Immunoprecipitation

The ORF encoding a GFP-binder [61] was cloned into pGEX-4T3 then transformed into *E. coli* strain BL21. The GST-GFP-binder fusion protein was induced with IPTG, purified using Glutathione sepharose beads (Amintra), eluted using soluble glutathione then dialyzed. The purified GST-GFP-binder protein was then used to affinity purify GFP-tagged proteins using Glutathione sepharose beads. In brief, cells were plated into four 10 cm dishes per condition, cultured for 24 hours followed by overnight induction of exogenous GFP-fused proteins using tetracycline. Cells were trypsinized, pooled and lysed in lysis buffer (0.1% Triton X-100, 100 mM NaCl, 10 mM Tris pH7.4, 1 mM EDTA, 1mM EGTA, 20 mM β-Glycerol, 10 mM NaF) then insoluble proteins removed by centrifugation. To purify GFP-tagged proteins, a 50% Glutathione magnetic bead slurry was washed in protein lysis buffer, added to the lysate along with 30 μg of GST-GFP-binder protein then incubated under rotation for three hours at 4°C. Beads were washed five times with lysis buffer then bound proteins eluted by boiling in SDS sample buffer. For input samples, 5% of the original supernatant was removed.

### Time-lapse imaging

For cell fate profiling, cells were seeded onto microclear 96 well plates (Greiner) then analyzed using an IncuCyte ZOOM (Essen BioSciences) equipped with a 20x objective, acquiring images every 10 minutes. Confluency measurements were performed using the IncuCyte ZOOM software. Real time quantitation of apoptosis was also performed on an IncuCyte ZOOM in conjunction with CellPlayer Kinetic Caspase-3/7 Apoptosis Assay Kit (EssenBioSciences) [32]. To determine mitotic timing and cell fate, MPEG-4 image sequences were analyzed manually, scoring 50 cells per condition from duplicate wells. Zero hours represents when imaging started unless indicated otherwise. Cell fate profiles were generated using Graphpad Prism 6 [10]. For fluorescent analysis of DLD-1 cells expressing the mCherry D-box fusion [10], cells were seeded in a microclear 96 well plate (Greiner) prior to RNAi transfections. For transient transfections of mCherry-Mcl-1 fragments and fluorescent tracking of AID-tagged proteins, cells were seeded into a 24 well microclear plate (Ibidi) then live cell imaging of fluorescent cells performed on an Axiovert 200 microscope with a 32x objective (Zeiss) enclosed in an environmental control chamber (Solent) with cells maintained at 37°C in a continuous flow of humidified CO_2_ [62]. Images were taken using a CoolSNAP HQ camera (Photometrics) and analyzed using Metamorph software (Molecular Devices).

### Quantitative polymerase chain reaction (qPCR)

RNA was extracted from cells using TRIzol-chloroform (Invitrogen) according to the manufacturers instructions, precipitated using 100% isopropanol then re-suspended in RNase-free water. Samples were DNase-treated and reverse transcribed using random hexamers, Reverse Transcriptase and RNase inhibitor (all Qiagen). Amplification of specific cDNAs was performed on an Mx3000P qPCR system (Agilent Technologies) using SYBR green (Qiagen) and the following primers: MULE (5′-GGGGTTATGACCCAAGAGGT-3′, 5′-CCCATCTCGAGACTCCTCTG-3′); GAPDH (5′-CCACCCATGGCAAATTCCATGGCA-3′, 5′-TCTAGACGGCAGGTCAGGTCCACC-3′). Analysis was performed using the delta CT method.

### Statistical tests

Cumulative frequency plots and statistical analyses (non-parametric Mann-Whitney U tests) were performed on Graphpad Prism 6. Box-and-whisker plots show the mean and the interquartile ranges.

## SUPPLEMENTARY FIGURES


