# Supplementary material for: Mcl-1 dynamics influence mitotic slippage and death in mitosis
Source: Oncotarget. 2016 Jan 12;7(5):5176–92. doi: 10.18632/oncotarget.6894 (PMC4868679; doi:10.18632/oncotarget.6894)
Supplement: Supplementary file 1 [file oncotarget-07-5176-s001.pdf]

SUPPLEMENTARY FIGURES

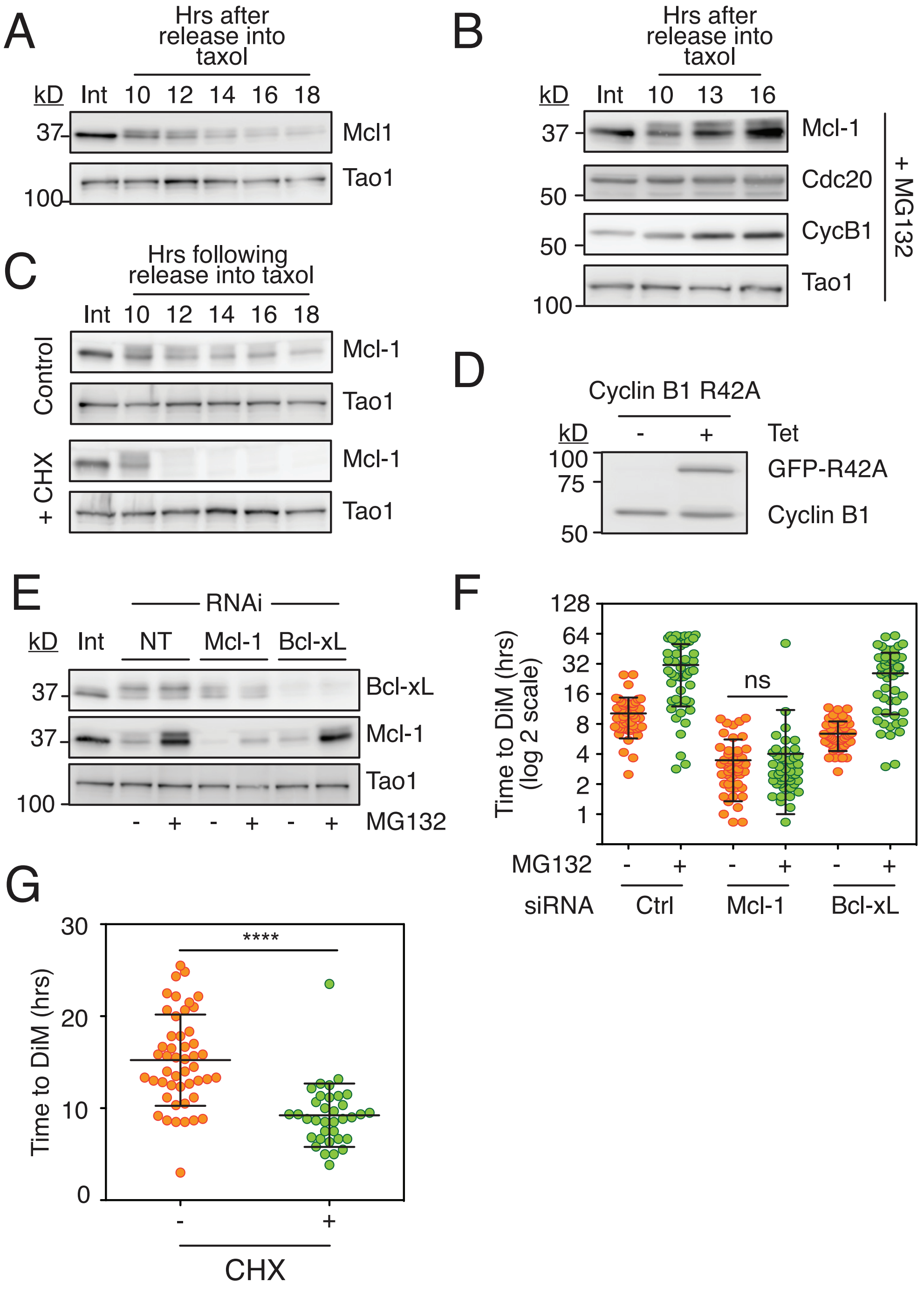

Supplementary Figure S1: Mcl-1 is synthesized and degraded in mitosis. A. Immunoblot of Mcl-1 levels after synchronized cells were released from thymidine block into 0.1 µM taxol and protein samples taken at indicated times. An interphase sample (Int) is shown for comparison. **B.** Immunoblot of Mcl-1 levels in RKO cells following MG132 treatment 10 hours after addition of taxol. **C.** Immunoblot showing Mcl-1 levels in RKO cells following cycloheximide treatment 10 hours after addition of taxol. **D.** Immunoblot showing endogenous Cyclin B1 and GFP-tagged Cyclin B1 R42A upon induction with 1 µg/ml tetracycline. **E.** Immunoblot of Mcl-1 and Bcl-xL protein after siRNA transfection, followed by exposure to taxol and MG132 for 6 hours. **F.** Time to death of cells depicted in Fig. 1B treated with MG132. Mann Whitney U test, ns $p > 0.05$. **G.** Time to death of cells depicted in Fig. 1B showing that cycloheximide accelerates mitotic apoptosis. Mann Whitney U test, **** $p < 0.0001$.

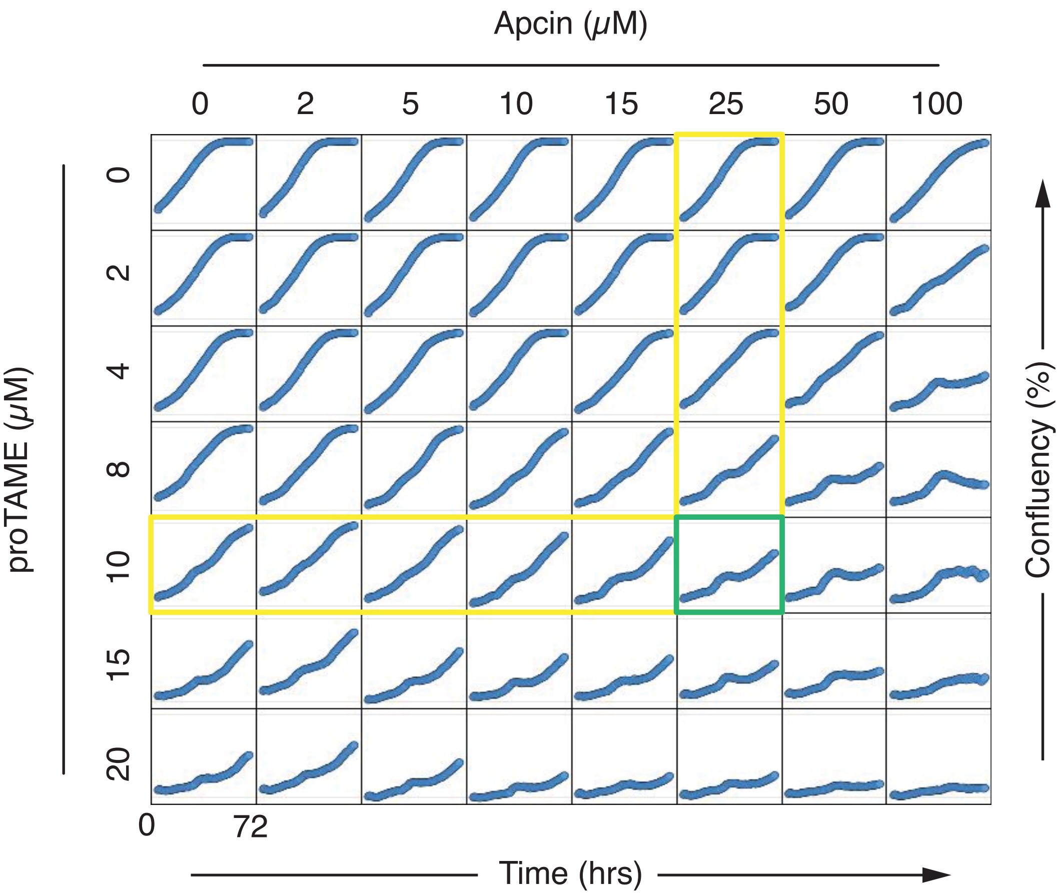

Supplementary Figure S2: Titration of APC/C inhibitors. Confluency measurements of uninduced RKO Cyclin B1 R42A cells over 72 hours treated with combinations of APC/C-Cdc20 inhibitors proTAME and Apcin.

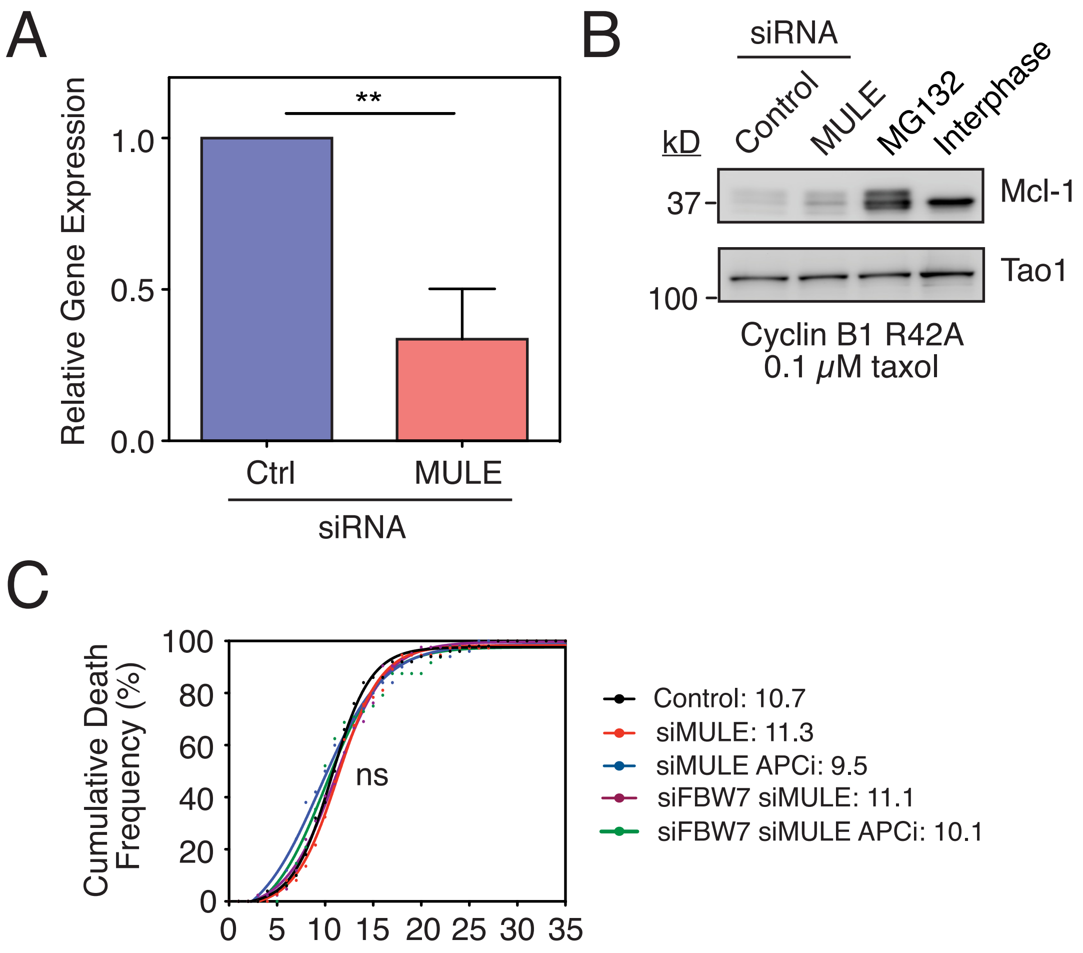

Supplementary Figure S3: Inhibition of E3 ligase MULE does not delay death in mitosis. A. qPCR showing reduced levels of MULE mRNA 24 hours following siRNA transfection. **B.** Immunoblot of Mcl-1 levels in RKO Cyclin B1 R42A cells following transfection with siRNAs targeting MULE. **C.** Cumulative death frequency graph of RKO Cyclin B1 R42A following trasfection of siRNAs targeting FBW7 and MULE then exposed to proTAME/Apcin. Mann Whitney U test, ns $p > 0.05$. In panels (B) and (C) Cyclin B1 R42A was induced with 1 μg/ml tetracycline and cells exposed to 0.1 μM taxol.

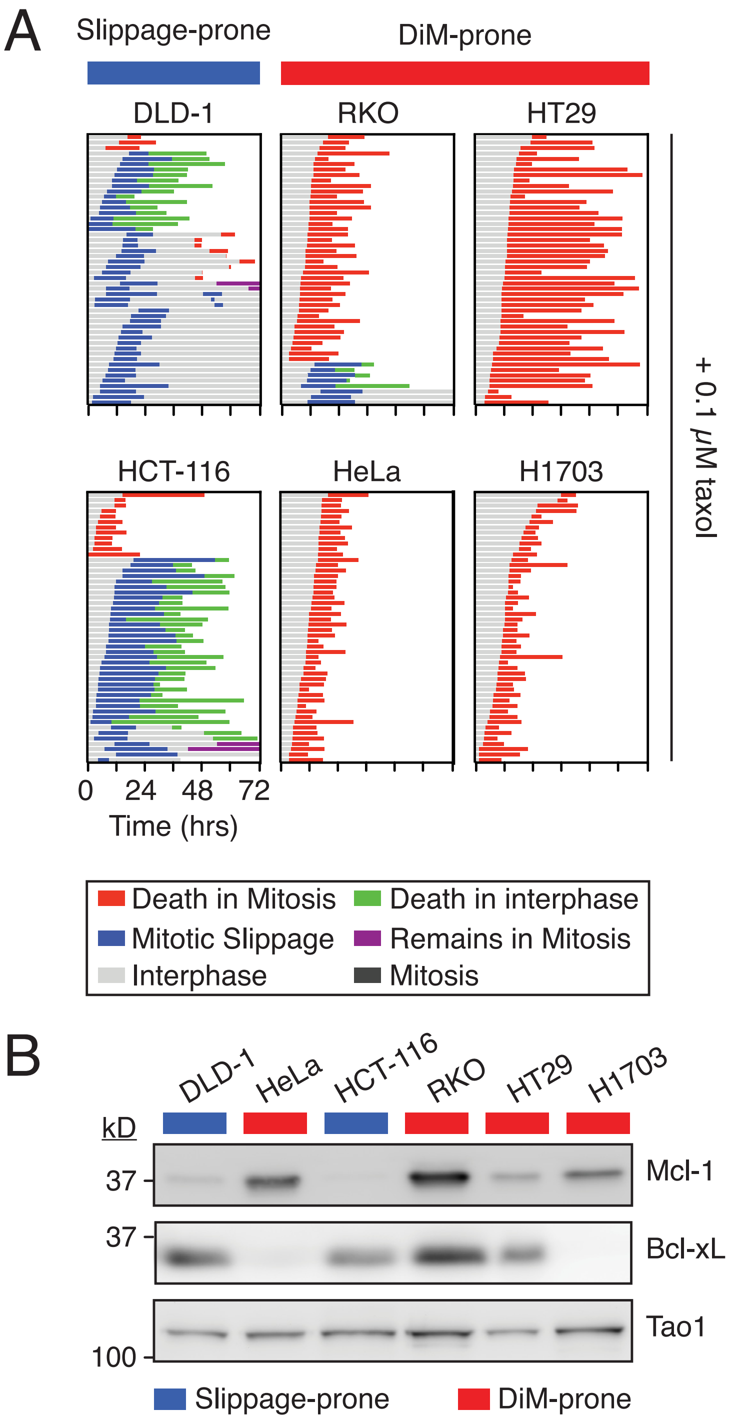

Supplementary Figure S4: Relative levels of Mcl-1 and Bcl-xL in slippage-prone and death-prone cell lines. A. Cell fate profiles of the indicated lines treated with 0.1 μM taxol showing that DLD-1 and HCT116 are slippage prone (blue) while RKO, HeLa, HT29 and H1703 are prone to death in mitosis (red). **B.** Immunoblots showing relative Mcl-1 and Bcl-xL protein levels in the six cell lines indicated.

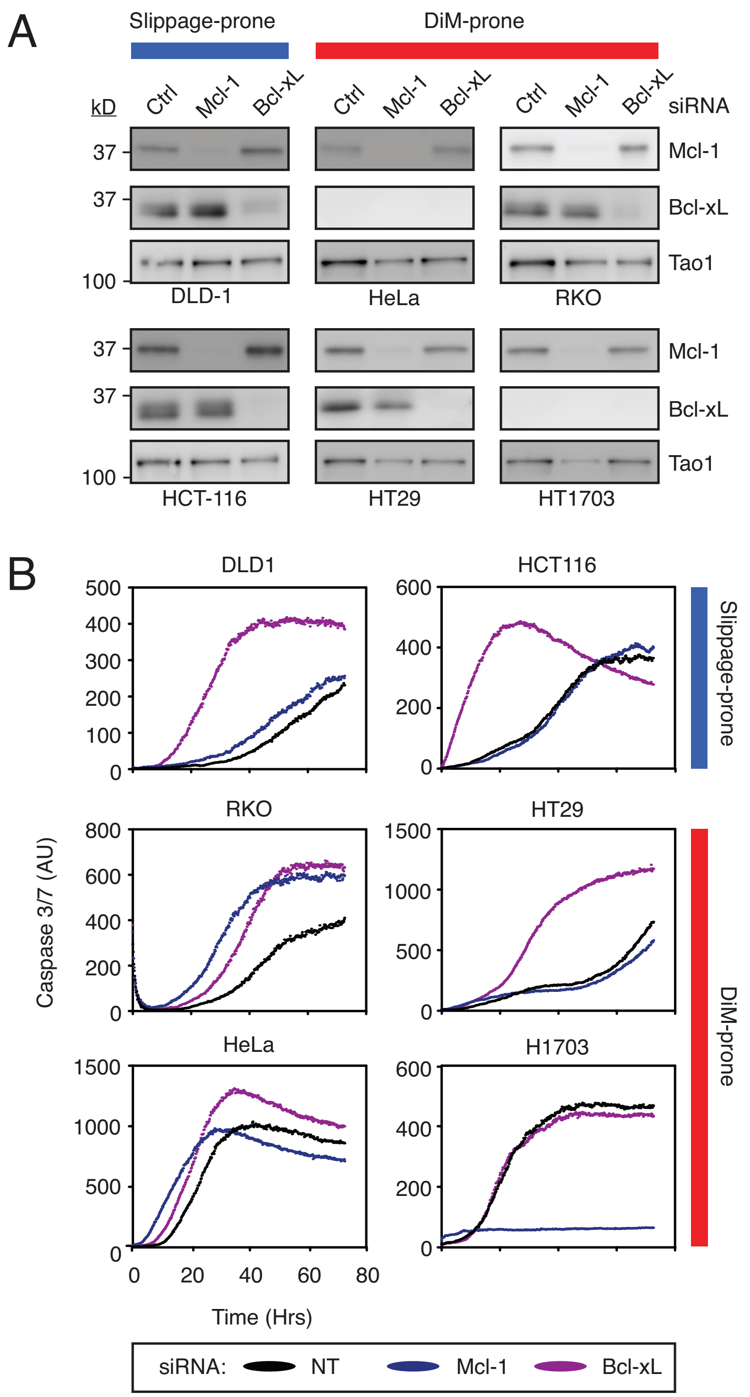

Supplementary Figure S5: Cell line specific effects of Mcl-1 and Bcl-xL depletion. A. Immunoblots showing RNAi-mediated inhibition of Mcl-1 and Bcl-xL 24 hours after siRNA transfection. **B.** Real-time apoptosis measurements of the indicated cells lines following RNAi-mediated inhibition of Mcl-1 or Bcl-xL and exposure to 0.1 μM taxol. Note that DLD-1, HCT116 and HT29 express relatively low levels of Mcl-1 and accordingly Mcl-1 RNAi has little effect. By contrast, these lines are sensitive to Bcl-xL inhibition. RKO cells express moderate levels of both Mcl-1 and Bcl-xL and inhibition of either enhances apoptosis, confirming functional overlap. H1703 express little Bcl-xL and accordingly are resistant to Bcl-xL RNAi. By contrast, H1703 cells are so sensitive to Mcl1 RNAi that most cells died before imaging started (not shown). Interpretation of the HeLa profile will require fate profiling.

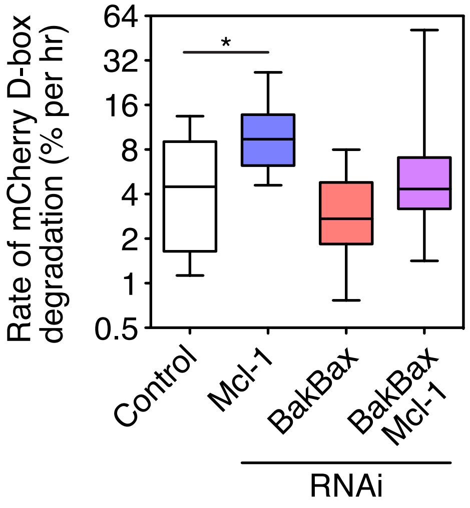

Supplementary Figure S6: Effect of Mcl-1, Bax and Bak depletion on Cyclin B1 degradation. Box-and-whisker plot quantitating the rate of degradation of a mCherry D-Box fusion in DLD-1 cells following RNAi-mediated inhibition of Mcl-1 and or Bax/Bak. At least 10 cells were analyzed per condition. Mann Whitney U test, * $p < 0.05$.

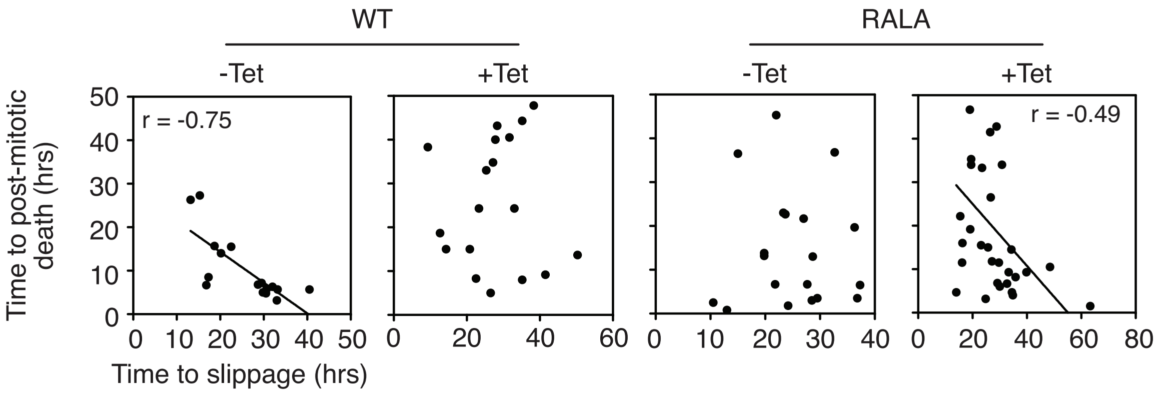

Supplementary Figure S7: Correlation analysis of DLD-1 Mcl-1 cell lines. Correlation analysis of the time to slippage versus the time to post-mitotic death in the cell populations shown in Fig. 7A.

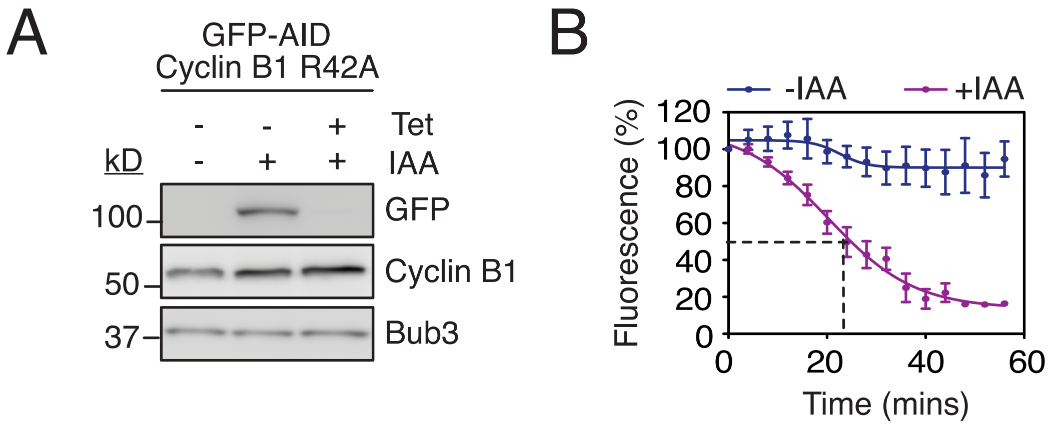

Supplementary Figure S8: Analysis of AID-tagged Cyclin B1 R42A. A. Immunoblot of Cyclin B1 and GFP protein levels 24 hours after addition of tetracycline and IAA to DLD-1 GFP-AID-Cyclin B1 R42A cells. **B.** Fluorescence intensity of DLD-1 GFP-AID-Cyclin B1 R42A cells treated with tetracycline. At zero minutes, IAA was added and cells arrested in mitosis were followed (n = 4).
